# Monthly Summary

**Published:** 1874-09

**Authors:** 


### MONTHLY SUMMARY.

*Experiments upon the Human Brain.*—The experiments were made upon a girl who had lost a portion of the cranium over a small space near the vertez, in consequence of an epithelioma of the scalp. For about two inches the dura mater was exposed and the pulsation of the brain visible. It was desired to ascertain the effect of irritation of the dura mater and cerebral substance through local faradization, galvanism, etc. Five needles guarded to near their points, used as electrodes, were thrust into the dura mater and brain-substance of either hemisphere. When the needles were passed into the dura mater no evidence of sensibility appeared. Faradization of the dura mater of one side caused muscular contractions of the opposite side, especially of the extensors of the extremities. The faradic reaction of the posterior lobes was tested by thrusting one needle into the brain while the other was placed in its vicinity in contact with the dura mater. A weak electric current caused painful tingling in the opposite extremities, dilatation of the pupils and muscular contractions of the opposite side. The strength of the current being increased, convulsive action upou the opposite side ensued, followed by frothing at the mouth, stertorous breathing and coma. The attack lasted twenty minutes.

The galvanic reaction of the posterior lobes was to have been tested, but rapid extension of the disease to the left hemisphere, with grave cerebral symptoms, precluded continuance of the experiments. The case soon resulted fatally. At the *post-mortem* no especial injury to the brain matter appeared to have been directly due to the needle-punctures. Their course was marked by lines of diffluent cerebral matter, but the surrounding tissue did not seem to have been affected by the lesion.—*Prof. Bartholou in American Journal of Medical Science.*

---

*Preservation from Hydrophobia.*—At the meeting of the French Academy of Sciences, on April 13th, M. Couley laid before it a memoir by M. Bourrell, a veterinary surgeon of Paris, entitled "A Complete Treatise on Rabies in the Dog and Cat, with a Method of Preserving one's self Against It." The means of preserving from rabies recommended by the author, consists in taking off the edge of the teeth of the animal with the aid of nippers or a file.(?) M. Bourrell had the daring to perform this operation of filing down the teeth of three dogs when they were in a condition of raging madness. Six dogs kept for experiment were then turned loose among the rabid animals, who attacked and bit them furiously, but without breaking the skin in any one of them. The dogs experimented on were watched during six months, and madness did not show itself in any one of the number. M. Bourrell, convinced that the blunted tooth of the dog could not penetrate through clothing, gave his hand, covered with a glove, to one of the mad dogs. "When," he says, "the dog released it the glove was intact, and the bite had only produced a deep impression." The same experiment repeated on dogs which were not rabid proved that the blunted tooth, however, violent the effort of the muscles of the jaw, would not penetrate the skin of animals, and affected the human epidermis in very exceptional cases only.—*Med. Record.*

---

*Treatment of Poisoning by Chloral.*—Dr. Albert Erlenmeyer discusses (*Der Praktishe Arzt*, Band xiv. 11) the best method of treating patients who, either by inadvertence or idiosyncrasy, have taken too large a dose of chloral. The symptoms of the toxic influence of this substance are—collapse, diminution of the frequency of respiration, which has been observed to be reduced to four in a minute ; injection of the conjunctiva, contraction of the pupil, blueness of the lips, dropping of the lower jaw and retracted tongue, whilst the pulse is in the early stage strong and slow, but subsequently becomes freqent and feeble, and ultimately scarcely perceptible. In more protracted cases, the face becomes pale, there is a tendency to fainting and vomiting rigors, disturbance of velutary movements, weakness of the lower limbs, and cramps in the calves of the legs, Erlenmeyer recommends, first, that the chloral should be removed from the stomach by emetics or the stomach-pump, or be much diluted with water, tea, or coffee ; secondly, that artificial respiration should be maintained ; and thirdly, that some antidote should be given. Erlenmeyer doubts the value of strychnia as recommended by Liebreich, since, although chloral is useful as an antidote to strychnia, it by no means follows that strychnia should be an antidote to chloral ; for we find that morphia is an antidote to atropia poisoning, but atropia is not an antidote in poisoning by morphia. He thinks musk might be tried, but is inclined to place most reliance on liquor ammoniæ subcutaneously injected. As a last resource, transfusion my be adopted.—*Practitioner.*

*Electricity for Chilblains.*—An Italian physician claims great success in the treatment of chilblains with the electro-magnetic apparatus. A current of medium intensity is passed through the diseased part for fifteen minutes, with great relief to pain. One or two sittings will cure—so it is said. The same treatment has been successfully employed in neuralgia of the testis, in which the pain was so excruciating, that the patient begged to have the gland extirpated. A case also is reported of successful treatment by the same means, of an obstinate lumbago which had resisted every other plan.—*Pacific Medical and Surgical Journal.*

---

*Cure for the Toothache*—Dr Henry T. Reynolds, of Baltimore, writes to the editor of the *Medical News* that, for eighteen months he has been using acetate of lead as a remedy for toothache. He finds it better than any of the numerous remedies proposed in the books, and in cases in which it is applicable, the relief is instantaneous. He advises the sufferer to apply from one to three grains to the cavity for a moment or two, then spit it out. It fails in fewer cases than any remedy that Dr. Reynolds ever tried, not more than eight per cent.

---

*Marine Glue.*—The *Journal of Applied Chemistry*, gives the following, which is said to be both firm and waterproof: 1 lb. of caoutchouc, cut into small pieces by means of a wet knife, dissolved in 4 gallons of wood naptha. The solution of the gum should be aided by frequent stirring, and will usually occupy 10 or 12 days. When this is completed 2 pounds of shellac are melted in an iron ladle, and one pound of the solution stirred in, and the glue poured out to cool upon slabs.

---

*Ether and Chloroform.*—" We are able to say that in the present state of science the medical man is responsible for every case of death occasioned by the application of ether, because a careful watching of the respiration is capable of preventing death, whilst the lethal effect of chloroform depends in part on individual predisposition, which the physician is unable to recognize."—*Prof. Schiff, of Florence.*

---

*Singular Death from Blood-poisoning.*—Dr. Weigel, a surgeon connected with the military hospital at Münster, was recently made dangerously ill, the result of inoculation with morbid matter absorbed at an autopsy through a slight wound in his hand. In performing an operation for his relief, another surgeon, Dr. Kruse, chanced to make a slight incision in his finger, by which means he himself became inoculated, and, after severe suffering, died.—*Boston Med. & Surg. Jour.*

